# The greenhouse gas emissions performance of cellulosic ethanol supply chains in Europe

**DOI:** 10.1186/1754-6834-2-15

**Published:** 2009-08-14

**Authors:** Raphael Slade, Ausilio Bauen, Nilay Shah

**Affiliations:** 1 Imperial Centre for Energy Policy and Technology, Centre for Environmental Policy, Imperial College London, London, UK; 2 Centre for Process Systems Engineering, Imperial College London, London, UK

## Abstract

**Background:**

Calculating the greenhouse gas savings that may be attributed to biofuels is problematic because production systems are inherently complex and methods used to quantify savings are subjective. Differing approaches and interpretations have fuelled a debate about the environmental merit of biofuels, and consequently about the level of policy support that can be justified. This paper estimates and compares emissions from plausible supply chains for lignocellulosic ethanol production, exemplified using data specific to the UK and Sweden. The common elements that give rise to the greatest greenhouse gas emissions are identified and the sensitivity of total emissions to variations in these elements is estimated. The implications of including consequential impacts including indirect land-use change, and the effects of selecting alternative allocation methods on the interpretation of results are discussed.

**Results:**

We find that the most important factors affecting supply chain emissions are the emissions embodied in biomass production, the use of electricity in the conversion process and potentially consequential impacts: indirect land-use change and fertiliser replacement. The large quantity of electricity consumed during enzyme manufacture suggests that enzymatic conversion processes may give rise to greater greenhouse gas emissions than the dilute acid conversion process, even though the dilute acid process has a somewhat lower ethanol yield.

**Conclusion:**

The lignocellulosic ethanol supply chains considered here all lead to greenhouse gas savings relative to gasoline An important caveat to this is that if lignocellulosic ethanol production uses feedstocks that lead to indirect land-use change, or other significant consequential impacts, the benefit may be greatly reduced.

Co-locating ethanol, electricity generation and enzyme production in a single facility may improve performance, particularly if this allows the number of energy intensive steps in enzyme production to be reduced, or if other process synergies are available. If biofuels policy in the EU remains contingent on favourable environmental performance then the multi-scale nature of bioenergy supply chains presents a genuine challenge. Lignocellulosic ethanol holds promise for emission reductions, but maximising greenhouse gas savings will not only require efficient supply chain design but also a better understanding of the spatial and temporal factors which affect overall performance.

## Background

The production of transport fuels from lignocellulosic biomass using so-called second-generation conversion technologies is not yet commercial. Multiple conversion pathways are being investigated around the globe, but dominant pathways have yet to emerge and business models have yet to be proven. Nevertheless, expectations are running high and there has been significant investment in research and demonstration by private companies and public sector organisations in the US, Europe and Asia. The production of ethanol from lignocellulosic biomass is one of the most promising options, and in 2007 the US Department of Energy provided more than US$1billion toward lignocellulosic ethanol (LE) projects, with the goal of making the fuel cost competitive at US$1.33 per gallon by 2012 [1]. The level of support provided by the European Union (EU) is far less, but is still significant (approximately US$68million in 2006 [2]). In addition to support for R&D and demonstration, LE, if it enters the market, would benefit from measures that seek to promote the use of currently available biofuels, that is, biofuels that are produced from agricultural commodities.

The political support for biofuels in the EU is, in part, predicated on their ability to reduce greenhouse gas (GHG) emissions [3]. Quantifying the GHG savings that may be attributed to biofuels, however, is problematic for two reasons: (i) biomass production systems are inherently complex, spatially disaggregated and diverse, and (ii) the definition of system boundaries and the allocation of co-product impacts are highly subjective. Differing subjective interpretations have fuelled an active debate about the environmental merit of biofuels and, consequently, about the level of policy support that can be justified. Politically, however, decisions must be made before all the uncertainties are resolved [4].

This paper focuses on the production of ethanol from lignocellulosic feedstocks in Europe, exemplified using data specific to the UK and Sweden. The aim was to compare a number of plausible supply chains for LE production, to identify which common elements gave rise to the greatest GHG emissions, and to investigate the sensitivity of total supply chain emissions to variations in these elements. It builds upon, and is complementary to, a previous paper that investigated the factors affecting the commercial viability of LE production in Europe [5].

To meet this aim an emissions model was developed that permitted the comparison of different process concepts at the supply chain level. This model used simplified descriptions of LE conversion processes, together with emission factor estimates for feedstocks and supply chain operations, to determine the sensitivity of GHG emissions estimates to changes in the supply chain.

This paper is presented in three parts. This first part describes the context, reviews previous GHG estimates, and outlines why performance assessments of bioenergy supply chains are particularly susceptible to subjective interpretation. The basis for supply chain emissions comparison is defined and the basic structure of the model is described. The second part describes the components of representative ethanol supply chains. It identifies generic values for the most important parameters affecting emissions performance, as well as the range of values that these parameters may take and the influence of alternative allocation methodologies on their interpretation. The last part presents a comparison and detailed sensitivity analysis, and identifies which supply chain components have the greatest influence on GHG emissions.

### The relative merit of alternative biofuels

The relative merit of biofuel production from different feedstocks has been the subject of many studies and much debate. Most studies have used the Life Cycle Assessment (LCA) methodology, or a variation on it, to quantify the environmental burdens arising from feedstock production, conversion processes and fuel distribution. The LCA methodology, although formalised by the International Standards Organisation (ISO14040), has a number of limitations [6,7]:

1. The definition of system boundaries, the allocation of impacts, and the choice of data sources are inherently subjective.

2. Good quality data may not exist, or may not be readily accessible.

3. Spatial and temporal resolution is lost.

4. Rebound effects, where environmental and cost efficiency improvements are cancelled out by greater consumption, are not considered.

These limitations are well recognised and have led to calls for greater consistency, transparency and coherence in LCA studies [8].

In an attempt to compare different biofuel supply chains on a robust basis and provide the consistency and transparency demanded, a number of influential meta-studies have been conducted. These studies have re-analysed previous LCAs, drawing the system boundary around an individual production plant and its feedstock supply chain [8-10]. The studies differ somewhat in approach but agree upon a general conclusion: cellulosic ethanol results in greater carbon savings (75 to 150 gCO_2_e.km^-1^) than wheat (15 to 110 gCO_2_e.km^-1^) or maize (40 to 60 gCO_2_e.km^-1^) but not necessarily as great as from Brazilian sugar cane (125 to 175 gCO_2_e.km^-1^).

This conclusion, however, does not stand uncontested. Two subsequent studies assert that the system boundaries should be expanded to include the consequential impacts – for example, land-use change impacts – that may result from increased demand for agricultural commodities and land [11,12]. They argue that these impacts have the potential to negate the benefits obtained from increased biofuel production unless the biofuels are produced from waste materials or on land with a low carbon-stock value that is not under productive use.

It is our view that biofuel systems are more susceptible to the shortcomings of the LCA method, and to differences in interpretation, than typical petrochemical processes working with standardised equipment and commodity feedstocks. This susceptibility arises because of the multi-scale nature of biomass supply chains. In particular:

1. Biomass feedstocks are varied in nature, low energy density, geographically dispersed, and their availability for fuel production is dependent on interactions with existing markets; moreover, data relating to agricultural practices is scarce.

2. Logistics may contribute significantly to the overall environmental impact.

3. Environmental and technical performance is highly dependent on the detailed process configuration and the level of integration with other systems, for example, district heating [13].

By identifying which LE supply chain components and allocation methodologies have the greatest impact on GHG emissions, and by assessing the interpretation of these impacts, this paper goes some way towards addressing these issues.

### The relationship between greenhouse gas emission projections, process development tools, and supply chain descriptions

GHG emission projections, like those for cost and commercial viability, are ultimately determined by the mass and energy balances of the conversion processes together with the structure of the feedstock-supply and product-distribution chains. Conversion processes for LE, however, have yet to be proven at commercial scale. Consequently, mass and energy balance estimates must be derived from flow sheeting models. These models are typically constructed to assist with process development and, although there are a number of good examples in the literature [14-17], they focus exclusively on the cost performance of alternative process designs. This emphasis on the conversion process ignores or sets as constant many of the factors that may influence overall supply chain cost performance. It also leaves the supply chain GHG performance unexplored.

Many of the components that make up feedstock-supply and product-distribution chains are already established; nevertheless, these chains are often poorly characterised and possess a high level of inherent variability. Whereas for cost projections, feedstock prices may reasonably be estimated by looking at pre-existing markets, the carbon emissions embodied in feedstocks cannot easily be divorced from the method of production. Consequently, a more detailed description of the feedstock supply is required. For agricultural commodities, for example, fertiliser use may have little impact on the market price but a large impact on embodied carbon emissions. Conversely, the product-distribution chain (percentage blend, level of subsidy and so on) has a large impact on the price obtained for ethanol when sold but little impact on emissions from combustion. (Ethanol has a lower volumetric energy density than gasoline but may be combusted more efficiently. The precise difference, in terms of MJ fuel per unit of useful work done, will depend upon an engine's compression ratio, drive cycle, percentage blend level, and so on, but is nonetheless small (± 5%) [18,19].)

### Developing a supply chain greenhouse gas model

#### Quantifying supply chain greenhouse gas performance

This analysis focuses on the carbon and energy intensity of the supply chain and only considers the direct energy consumption necessary to produce, transport and transform material inputs, and the GHG emissions associated with these operations. The energy required to construct, maintain or replace capital equipment whose lifetime far exceeds that of the fuel produced was not considered. This assumption is consistent with the Concawe well-to-wheels methodology [20].

The end point of the supply chain was considered to be the embodied GHG emissions of ethanol when delivered to the pump. The starting point was the production of biomass feedstocks. Emissions are reported as the mass of carbon dioxide equivalents per GJ ethanol, calculated from the summation of embodied carbon emissions across the supply chain. The advantage of this unit is that it is readily comparable with the carbon emissions associated with combusting gasoline (74.2 kgCO_2_e.GJ^-1^) [21].

#### Model description

**Figure 1:**
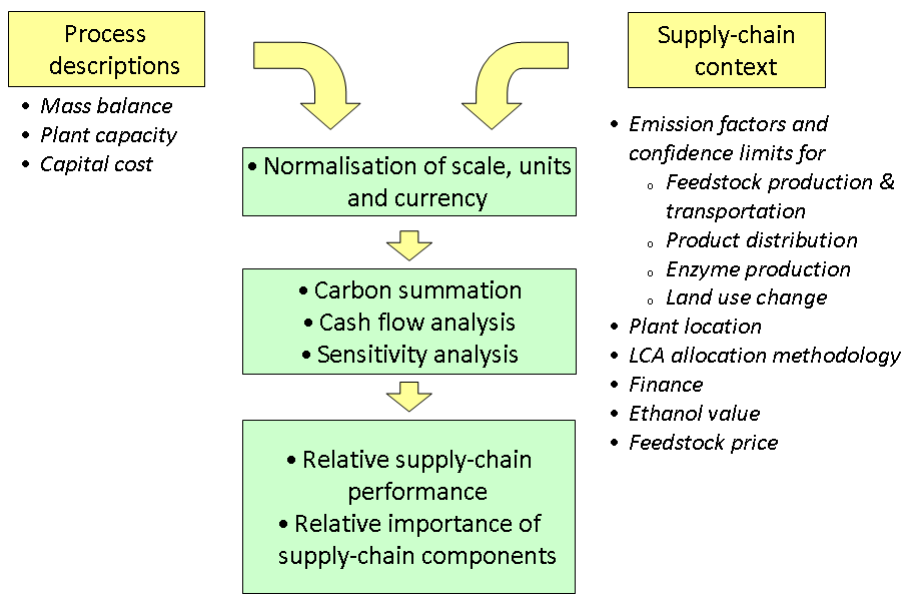
**Combined greenhouse gas and cost model schematic**.

The supply chain GHG model developed here is a spreadsheet-based tool incorporating a macro-driven sensitivity analysis. The model is an adaptation and extension of a cost model that has already been described in detail [5]. The rationale for extending an existing cost model was straightforward: the elements of a LE supply chain which determine its GHG performance are closely related to the elements which determine its cost performance. The combined model is shown schematically in Figure 1. The model inputs are twofold: firstly, descriptions of the conversion plant and process (mass and energy balance, plant capacity) and secondly, descriptions of the supply chain context (feedstock production, transportation and distribution and associated emission factors). The extended model permits the simultaneous evaluation of supply chain cost and GHG performance. This paper, however, focuses solely on the GHG results.

#### Verification of model inputs

Our model is, in essence, a summation of embodied carbon estimates in which numerous assumptions and decisions are incorporated. It cannot be tested empirically, but to ensure its overall validity, multiple estimates for input parameters were considered, taken from a wide range of sources. Data and assumptions were discussed with experts and were moderated accordingly. The sensitivity analysis embodied within the model also helped identify those areas where greatest precision was necessary.

## Methods

Our analysis focuses on a limited number of supply chains. Specifically, we consider ethanol produced from softwood or straw, using a dilute acid or enzymatic conversion process. These supply chains are amongst those with the greatest potential within Europe. Softwood and forest fuels are of interest because of their abundance (approximately 411 TWh.y^-1 ^[22]) in Northern Europe and the low input intensity of silviculture compared with agriculture. Straw is of interest because of its abundance as a co-product of cereal production (approximately 63 to 227 TWh.y^-1 ^[23]), and relatively low cost. For each supply chain, representative values for the most important parameters affecting embodied carbon emissions were identified and normalised.

### Characterising feedstock parameters

To construct base-case supply chains for comparison, estimates of the embodied GHG emissions for biomass feedstocks were identified in the academic and grey literature for Sweden and the UK. To enable estimates to be compared on a similar basis, each estimate was normalised using the following assumptions:

1. The feedstock supply chain was assumed to be composed of three generic operations:

1.a. Production and forwarding to a roadside collection point.

1.b. Transport from the roadside collection point to the processing plant.

1.c. Size reduction, where required, in order that the biomass was in a form acceptable to the plant.

2. The conversion plant was assumed to only receive chips, or in the case of the straw process, bales.

3. Biomass was transported in the densest form possible; for example, for logs, chipping at the plant was given preference to chipping at the roadside. (Chipping at the plant is more efficient and, although biomass transport is not necessarily limited by the bulk volume, transporting low-density biomass may require specialist vehicles.)

4. Where estimates did not include transport (and/or size reduction), a constant carbon intensity was assumed for each operation, its value determined by the biomass form (see Table 1, [24-27]).

**Table 1:** Emission assumptions for transportation and size reduction operations.

**Operation**	**Biomass form**	**Average Carbon Emissions from fossil fuel kgCO_2_e.odt^-1^**
Transport from roadside	Softwood logs^a^	4.6
	
	Softwood chips^b^	7.2
	
	Softwood bundles^b^	7.2
	
	Straw bales^b^	6.6

Size reduction^c^	Softwood (all forms)	8.5

These values were used to normalise feedstock emission estimates.

^a^Estimated emissions from fuel consumption assuming a 107 km trip – the average transport distance for logs in Sweden [24,25]. ^b^Estimated emissions from fuel consumption assuming 50 km trip [26]. ^c^Hammermill – 12 month operation window [27].

5. GHG emissions from biomass production were assumed not to vary with the quantity demanded.

6. Quantities were converted to oven-dry-tonne (odt) equivalents.

Total feedstock emissions were then calculated as the sum of emissions at roadside, from transport and from size reduction. It was found that the level of variation between estimates for similar resources was comparable to the level of variation between different resource types. It was therefore decided to use mid-point, high and low values as the principal inputs to the model. The mid-point corresponded to the geometric mean and the high and low estimates corresponding to the 15^th ^and 85^th ^percentiles respectively (that is, approximately one standard deviation from the mean), see Table 2, [28]. An exception was made in the case of the GHG emissions associated with straw production because the difference between minimum and maximum estimates was nearly two orders of magnitude, depending upon whether it was assumed that the nutrient value of the straw was replaced with inorganic fertilisers. To reflect this, only high and low values were used, and the base-case supply chains assumed the lower value.

**Table 2:** Normalised embodied carbon estimates for biomass feedstocks

**Feedstock** (delivered to the plant as chips/bales)	**Carbon emissions** (kgCO_2_e.odt^-1)^		
	
	**Low**	**Mid**	**High**
**Softwood^a^**	24.7	46.0	85.7

**Straw**	2.6	N/A	177.6

^a^The values shown are derived from literature values for softwood products that fall within the following ranges: logs: 25 kgCO_2_e.odt^-1^; forestry residues: 27 to 132 kgCO_2_e.odt^-1 ^and energy crops: 29 to 73 kgCO_2_e.odt^-1 ^[8,26,28].

It should be recognised that these base-case assumptions represent a substantial simplification. Relatively few data points were available, transport distances were assumed to be constant and the estimates exclude emissions that may arise as a consequence of increasing biomass utilisation. Nevertheless, the level of resolution corresponds with the quality and availability of data and is consistent with the analytical methods underpinning a number of UK Government reports, including the recent UK Biomass Strategy [29]. A more in-depth treatment of logistics and consequential impacts is described and evaluated below.

### Characterising the conversion process

Reference-case conversion processes were adapted from Aspen Plus™ models of a softwood, 25 odt.h^-1 ^(approximately 55 M.l.y^-1^ethanol) stand-alone facility, developed by the University of Lund and validated by laboratory scale experiments [17]. The enzymatic plant employed single-step steam-and-SO_2_-catalysed pre-treatment followed by simultaneous saccharification and fermentation using commercially purchased enzymes and yeast produced in the plant; surplus solid fuel (that is, lignin not required for process heat generation) was exported and sold. The dilute acid plant was similar to the enzymatic process, except that separate hydrolysis and fermentation was undertaken using a two-stage acid catalysed pre-treatment and hydrolysis step. Detailed mass balance data are shown in Table 3.

**Table 3:** Mass balance and assumptions for reference-case enzymatic and dilute acid conversion processes.

**Feedstock/co-product** (units)		**Mass balance**									
		**Enzymatic process^b^**					**Dilute acid process^b^**				
		
		**Input** (unit.odt^-1^)	**Output** (unit.odt^-1^)				**Input** (unit.odt^-1^)	**Output** (unit.odt^-1^)			
			**Ethanol**	**CO_2_**	**Solid fuel**	**Waste** (solid + liquid)		**Ethanol**	**CO_2_**	**Solid fuel**	**Waste** (solid + liquid)
	
**Biomass**											
Hexose	kg	620	219	245		156	620	173	190		257
Pentose		60				60	60				60
Lignin		280			252	28	280			273	7
Other		40				40	40				40
**Chemicals**											
SO_2_	Kg	15.48				15.48	0.00				
H_2_SO_4_		0.00					63.20				63.20
NaOH (50%)		28.96				28.96	28.96				28.96
NH_3 _(25%)		2.36				2.36	1.68				1.68
H_3_PO_4 _(50%)		0.52				0.52	0.36				0.36
Defoamer		0.56				0.56	0.44				0.44
(NH_4_)2PO_4_		2.76				2.76	2.60				2.60
MgSO4.7 H2O		0.12				0.12	0.12				0.12
Enzymes	10^6 ^FPU^a^	9.36				9.36	0.00				
Electricity-buy	MWh	0.18					0.18				
Cooling water	m^3^	72.48					65.44				
Process water	m^3^	3.36				3.36	3.20				3.20

^a^Filter paper unit. ^b^The reference processes were adapted to include pentose fermentation using the following assumptions: the ethanol and CO_2 _yield from pentose sugars was assumed to be 50%, reflecting the fact that the recovery of pentoses after pre-treatment is lower than for hexoses [17]; the solid fuel yield and process heat requirement was unaffected; the flow of chemicals was the same as the respective softwood enzymatic or dilute acid process. The reference processes were adapted to use straw as a feedstock using similar assumptions: the ethanol and CO_2 _yield from hexose sugars was assumed to be the same as for the respective softwood enzymatic, or dilute acid, reference process; the solid fuel yield and process heat requirement was unaffected; the flow of chemicals was unaffected [5].

### Alternative allocation methodologies

The methodology used to apportion supply chain GHG emissions between co-products affects the interpretation of emissions attributable to ethanol. The inputs to the supply chain considered here were biomass and fossil-fuel derived inputs: diesel, fertiliser, and so on. The outputs were ethanol, surplus solid fuel or electricity (obtained from lignin residues), CO_2 _from fermentation and the combustion of biomass residues, and CO_2 _from the consumption of diesel, production of fertiliser, and so on. (It should be noted that the production of biofuels may give rise to GHG emissions from a variety of other sources, for example, N_2_O from fertiliser and CO_2 _from the oxidation of organic matter in soil; these potential sources were included in the analysis only insofar as they were included in the literature estimates of the GHG emissions associated with biomass production.)

**Figure 2:**
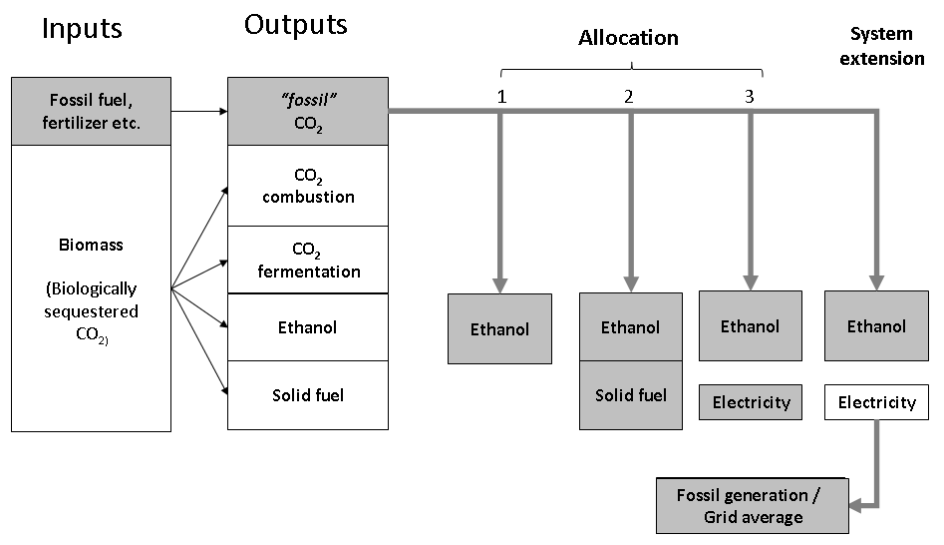
**Supply chain inputs, outputs, and greenhouse gas allocation methods**.

In the summation of impacts, the emissions from fermentation and the combustion of residues and principal co-products (ethanol and solid fuel) were excluded. This is because they release CO_2 _that was biologically sequestered in the plant material and therefore make no net contribution to the GHG balance. The other sources of GHG emissions, here labelled as 'fossil CO_2_' emissions, were then attributed to the ethanol and solid fuel co-products using one of four alternative methods. The first method allocated all fossil carbon emissions to the ethanol alone. The second assumed that surplus solid fuel was exported, and apportioned the fossil carbon emissions between the ethanol and solid fuel on the basis of energy content. The third method assumed that the solid fuel was transformed into electricity at the plant and that this electricity was then exported. (Again, fossil carbon emissions were apportioned between ethanol and electricity on the basis of energy content.) The last, substitution (system extension) methodology, assumed that the solid fuel was transformed into electricity at the plant and that this displaced grid electricity, thereby generating a carbon credit which partially offset the emissions allocated to ethanol. The supply chain inputs, outputs, and the four alternative allocation methods are shown graphically in Figure 2. It should be noted that none of these alternatives is novel and they are a small subset of allocation methods in common use. The objective here is to illustrate how selecting a particular allocation methodology affects the interpretation of results, not to present a new methodology. For a summary of the arguments for and against different allocation methodologies in relation to biofuel GHG reporting, see Bauen et al. [27].

### Electricity production

Electricity is a significant input to the conversion process. If this electricity is imported from the grid, its carbon intensity (assuming average grid emissions) varies significantly between European countries, depending upon the generation mix. To account for these differences the following average carbon intensities were used: EU27 (340 kgCO_2_e.MWh^-1^), UK (472 kgCO_2_e.MWh^-1^), Sweden (44 kgCO_2_e.MWh^-1^) [30].

### Modelling enzyme production

**Figure 3:**
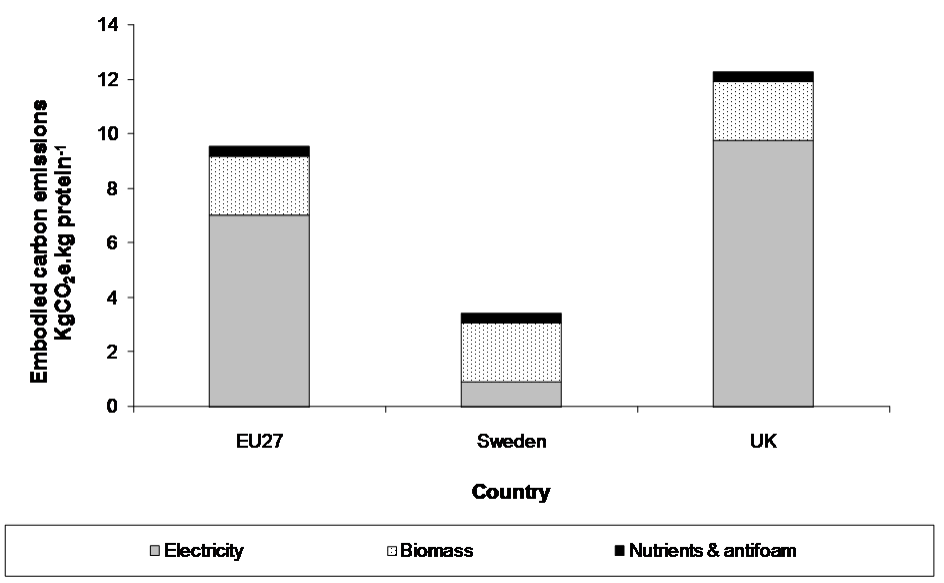
**Estimated greenhouse gas emissions from cellulase manufacture**.

Enzyme manufacture is known to be energy intensive, but no data was available about carbon emissions from cellulase production. To derive a cellulase-specific estimate, a simple model for off-site enzyme production was developed from the cellulase productivity assumptions described in the National Renewable Energy Laboratory's 1999 design report [14]. This model considered only the fermentation stage, assumed the use of grid electricity, and assumed that no GHG credit was available for the lignin residue co-produced with the enzymes. Detailed assumptions are summarised in Table 4[31,32]. Results for Sweden, the UK and the EU27 average are shown in Figure 3. It can be seen that electricity (used for agitation and air sparging) is one of the largest contributors to carbon emissions and that its precise contribution varies greatly with location. The overall results are consistent with carbon emission estimates for commonly produced amylase, phytase and protease enzymes (1 to 10 kgCO_2_e.kgprotein^-1^) [33].

**Table 4:** Cellulase manufacture assumptions

**Process assumptions**			**Emission assumptions**
**Inputs and parameters**	**Unit**	**Quantity^a^**	**kgCO_2_e.kg protein^-1^**
Fermentor volume	m^3^	1000	
Active volume	%	80%	
Cellulase productivity	FPU.l^-1^.h^-1^	75	
Cellulase activity	FPU.g protein^-1^	600	
Residence time	h	160	
Agitation power requirement	W.m^-3^	400	
Air sparge power requirement^e^	W.m^-3^	2183	
Biomass feedstock composition: cellulose/lignin	% (dry basis)	43%/28%	
Initial cellulose concentration	%	4%	
(NH_4_)2SO_4_	g.l^-1^	1.4	1.17^b^
KH_2_PO_4_	g.l^-1^	2	1.11^b^
MgSO_4_.7H_2_O	g.l^-1^	0.3	0.56^b^
CaCl_2_.2H_2_O	g.l^-1^	0.4	0.56^b^
Tween 80	g.l^-1^	0.2	0^c^
Corn oil anti foam	vol.vol^-1^	0.001	3.51^d^

^a^NREL 1999 design report [14]; ^b^estimated from GREET [31]; ^c^assumed to be negligible; ^d^assumed to be the same as rapeseed oil: LCAfood database [32]; ^e^assumes air sparge rate: 0.577 v.v^-1^.min^-1^; compressor capacity:630.6 m^3^.min^-1^; motor power: 2983 kW.

An alternative scenario to off-site enzyme production was also developed. This alternative, 'auto-generation' scenario, assumed that electricity was produced from the solid fuel co-product and used for enzyme manufacture on the same site as the ethanol plant, thereby reducing the quantity of electricity purchased. This scenario was modelled by adapting the mass balance for the enzymatic process so that the residual solid fuel from ethanol production, together with the lignin residue from enzyme manufacture, was converted to electricity at an efficiency of 30%. The quantities of electricity imported, and solid fuel exported, were correspondingly reduced.

### Modelling transport logistics

The base-case supply chains described above assumed fixed transport distances. To investigate the contribution of transport to overall feedstock carbon emissions in greater depth, a further set of supply chains was developed using a simple logistics model to estimate emissions from transport as a function of the quantity of feedstock demanded, the spatial density of the resource and the bulk density of the biomass.

For these supply chains, transport emissions were estimated as the emissions per *km.ton *multiplied by the total number of *km.tons *required for one year's operation. The total number of *km.tons *was modelled using a method adapted from Dornburg and Faaij [34]. This method assumed:

1. The average distribution of biomass collection points was constant and could be characterised as a fixed distribution density – *ρ (odt.km*^-2^).

2. Biomass for one year's operation – *m *(odt) – was supplied from a circular area with marginal radius – *r' (km) *– to a plant at the centre. Thus the marginal radius increased with the square root of the quantity of biomass demanded.

3. Road tortuosity was taken into account using an average winding factor (2^0.5^).

The total number of *km.tons *(*rm*_*tot*_) for one year's operation is thus given by the integral of the marginal radius with respect to mass, multiplied by the road tortuosity: *rm*_*tot *_= (2/3)^3/2^*.m*^3/2^*.(∏.ρ)*^-1/2^.

Transport was assumed to be by truck. The choice of vehicle was determined by the form of the biomass: logs were transported in wagons; chips, sawdust, and so on were either transported in tipping or moving floor trailers. The emissions per *km.ton *transported were estimated for logs, bundles, chips and bales according to the type of vehicle used and the density of the biomass. Emissions were assumed to arise solely from the use of diesel and were therefore directly proportional to the transport distance. (This assumption is consistent with the JEC well-to-wheels methodology [20].) Detailed logistics assumptions are summarised in Table 5[35].

**Table 5:** Logistics assumptions

		**Unit**	**Straw^c^**	**Softwood^b^**		
**Biomass parameters**	Form		Bales	Logs	Bundles	Chips
	Bulk density	odt.m^3^	0.11	0.462	0.251	0.219
	Distribution density	odt.km^-2^.yr^-1^	100	0.6	0.43	0.43

**Logistics parameters^a^**	Unit capacity	m^3^				100
	Daily availability	hr.day^-1^	18			
	Annual availability	day.yr^-1^	261			
	Vehicle speed	Km.h^-1^	50			
	Terminal time	hr.trip^-1^	2			
	Fuel economy	Km.l^-1^	2.7			

^a^[25]; ^b^[34]; ^c^[23] (GIS-based assessment of cereal straw energy in the European Union – average value for East Anglia, UK).

### Consideration of consequential impacts and land-use change

GHG emissions may arise from consequential impacts, the most important of which are arguably direct and indirect land-use change. Direct land-use change may occur if previously uncultivated land is used to produce biomass feedstocks; if the converted land had a high carbon stock value (for example, if it was forested) the GHG emissions from clearance and conversion may be significant. Indirect land-use change impacts may arise if increasing demand for biofuels increases commodity prices or displaces the production of other agricultural crops, and this, in turn, causes uncultivated land to be converted to agricultural production.

The Intergovernmental Panel on Climate Change (IPCC) provides guidance on the estimation of direct impacts (for example, the conversion of forest or grassland to annual or perennial biofuel crops) based on climate zone, ecological zone and soil type [36]. In the case of by-products (for example, straw) and managed forestry which continues to be managed (for example, softwood in northern Europe), the direct impacts, following IPCC 'tier 1' guidance, are nil.

Yet the science, and convention, for determining indirect impacts is in its infancy. To estimate the impacts from indirect land-use change, the UK Renewable Fuels Agency identifies two contrasting approaches: partial equilibrium modelling [12] and the use of indirect land-use change (ILUC) factors [37,38]. Both have been applied to first generation biofuels to investigate possible displacement effects resulting from the use of food crops for biofuels, but the indirect effects from the increased use of forest products (and residues such as straw) have received less attention.

In the absence of specific data, and consistent with our objective to show the relative importance of changes in the supply chain, the following assumptions were used to illustrate the potential effect, of including consequential impacts:

1. For softwood, consequential emissions were assumed to arise from an equivalent mass of short rotation coppice (SRC) grown on arable land, which in turn results in land elsewhere being converted to arable usage. Using an average yield of 10 odt.Ha^-1^.yr^-1 ^for SRC, and Fritsche's estimate for the conversion of high carbon content natural systems to arable land (4000 kgCO_2_e.Ha^-1^.year^-1^) [39] these assumptions yield a rough estimate of 400 kgCO_2_e.odt^-1^.

2. For straw, consequential emissions were assumed to be the same as the nutrient replacement value: 177.6 kgCO_2_e.odt^-1 ^(that is, the 'high' estimate in Table 2).

### Labelling individual supply chains

Many hundreds of supply chain permutations are possible. To clearly distinguish individual permutations the following labelling scheme is used:

**Feedstock: **Softwood = **Spruce**, Straw = **Straw**

**Feedstock emissions: **High = **(HC)**, Medium = **(MC)**, Low = **(LC)**

**Process: **Dilute acid = **DA**, Enzymatic = **EH**, Pentose fermentation = **p**

**Capacity: **25 odt.h^-1 ^= **C(25)**

For example, softwood with a low level of embodied carbon emissions, processed using an enzymatic process including pentose fermentation, in a plant with a capacity of 25 odt.h^-1^, would have the label: **Spruce(LC)-EHp-C(25)**.

## Results and discussion

### Comparison of base-case supply chains

**Figure 4:**
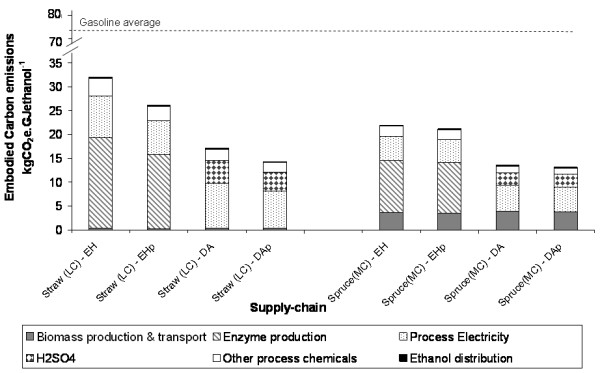
**Carbon emissions from ethanol production for base-case supply chains**. Greenhouse gas emissions arising from the use of fossil fuels, fertiliser, and so on, are apportioned between ethanol and solid fuel on the basis of energy content.

This section presents a comparison of LE supply chains developed from the components described above. The objective was to identify which elements have the greatest impact on embodied carbon emissions. The base-case chains which are compared are defined in Table 6. The embodied carbon emissions for each chain are shown in Figure 4.

**Table 6:** Base-case lignocellulosic ethanol supply-chains

**Supply chain label**	**Feedstock embodied emissions** **kgCO_2_e.odt^-1^**	**Electricity embodied emissions**	**Allocation method**	**Transport distance**

Straw-DA				
				
Straw-EH				
	Low (2.6)			
Straw-EHp				
				
Straw-DAp			All fossil carbon	
			emissions allocated to ethanol and solid fuel	Static average
Spruce-EH		EU 27 average		
				
Spruce-EHp				
	Med (46.0)			
Spruce-DA				
				
Spruce-DAp				

All the base-case chains lead to substantially reduced carbon emissions relative to gasoline (56 to 82% reduction). For enzymatic hydrolysis chains, the greatest contribution to emissions comes from enzyme production (50 to 60%), followed by electricity consumption in the conversion process (22 to 28%). For the acid hydrolysis chains, the greatest contribution to emissions comes from electricity consumption (40 to 55%). For the softwood chains, biomass contributes approximately 16 to 30%, whereas for the base-case straw chain the contribution is negligible. In all cases the emissions associated with other process chemicals are small (4 to 8%) and the emissions associated with transport and distribution are negligible (0.5 to 1.5%). Chains utilising pentose fermentation all show lower emissions than their non-pentose fermenting counterparts, demonstrating the advantage that may be obtained from incremental improvements in ethanol yield.

### The impact of allocation and location decisions

**Figure 5:**
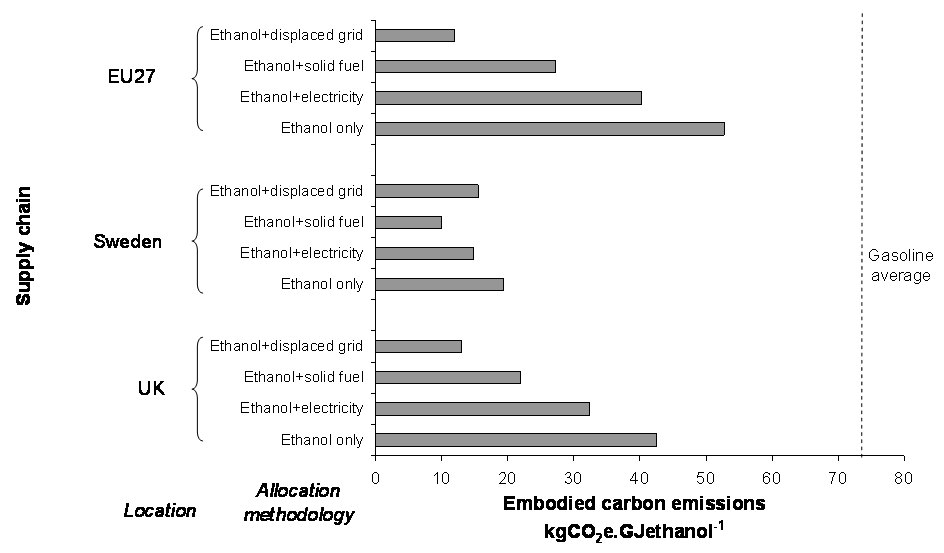
**The variation in embodied carbon emissions estimates for alternative allocation methods and locations**. The example shown is for the spruce enzymatic hydrolysis supply chain (Spruce-EH).

Figure 5 shows how the emissions estimates, and their interpretation, vary depending upon the allocation methodology selected and the location of the plant. The results shown are for the softwood enzymatic hydrolysis supply chain (Spruce(MC)-EH), but similar relationships can be demonstrated for the other supply chains.

It can be seen that allocating emissions to both ethanol and co-products reduces the emissions attributable to ethanol in all cases. With this method, a supply chain located in Sweden yields a greater carbon saving relative to gasoline (73 to 86% reduction) than the same chain located in the UK (28 to 63% reduction) or assuming the EU27 average electricity mix (42 to 70% reduction). This difference reflects the greater carbon intensity of electricity in the UK and EU27 compared with Sweden.

Using the substitution methodology, ethanol appears to have greatly reduced emissions compared with gasoline (74 to 84% reduction), but the impact of location is reversed: a chain located in Sweden appears worse than one located in the EU27 or UK. This is because the carbon credit obtained by displacing relatively clean Swedish grid electricity is approximately one tenth of that obtained by displacing UK or EU27 electricity; that is, the more carbon intensive the electricity system, the greater the apparent saving the substitution methodology will demonstrate.

It is arguable that the substitution method best reflects the situation on the ground for a specific plant. The disadvantage, however, is that expanding the system to include displaced electricity production (or other products) requires knowledge of the local situation. The products that are displaced may also change with variations in relative market price; consequently, generalisation from a specific instance may not be justified. Allocation on the basis of energy content avoids this problem to a certain extent, but it may be argued that this approach provides a less accurate reflection of the net impact of the bioethanol production system in the economy. Again, the issue of how to draw consistent boundaries arises because the solid fuel may be burnt directly or transformed into secondary products.

### Comparing offsite and onsite enzyme production

**Figure 6:**
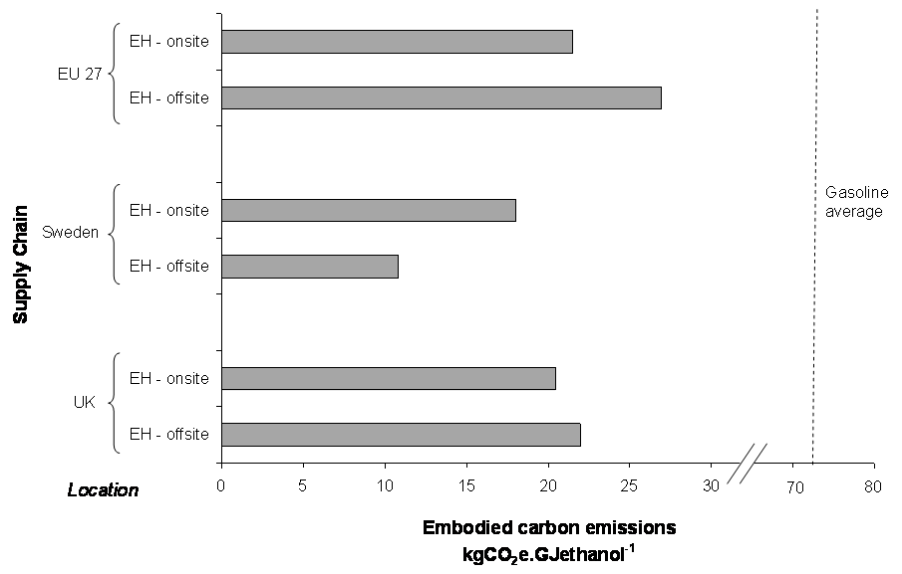
**The variation in embodied carbon emission estimates for alternative locations and onsite versus offsite enzyme production**. The example shown is for the spruce enzymatic hydrolysis supply chain (Spruce-EH); all fossil carbon emissions are allocated to ethanol and residual solid fuel in proportion to their energy content.

Figure 6 shows how the emissions estimates for the spruce enzymatic hydrolysis supply chain vary depending upon whether enzymes are produced onsite or offsite. It can be seen that onsite production leads to an apparent saving compared with offsite production where the electricity being displaced has a high carbon intensity (UK and EU27), but not where the displaced electricity has low carbon intensity (Sweden). This result should not be unexpected because onsite enzyme manufacture is analogous to the system extension allocation methodology described above. Not all the savings due to onsite enzyme production, however, are an artefact of location and allocation methodology: the inclusion of lignin residues from enzyme manufacture in the mass balance for ethanol production reduces the emission estimates by a small proportion (approximately 6%) compared with the base-case. It is also possible that an integrated flowsheet model could reveal further savings: for example, onsite production may allow emissions associated with purifying, drying and transporting the enzymes to be avoided altogether.

### The impact of transport emissions

**Figure 7:**
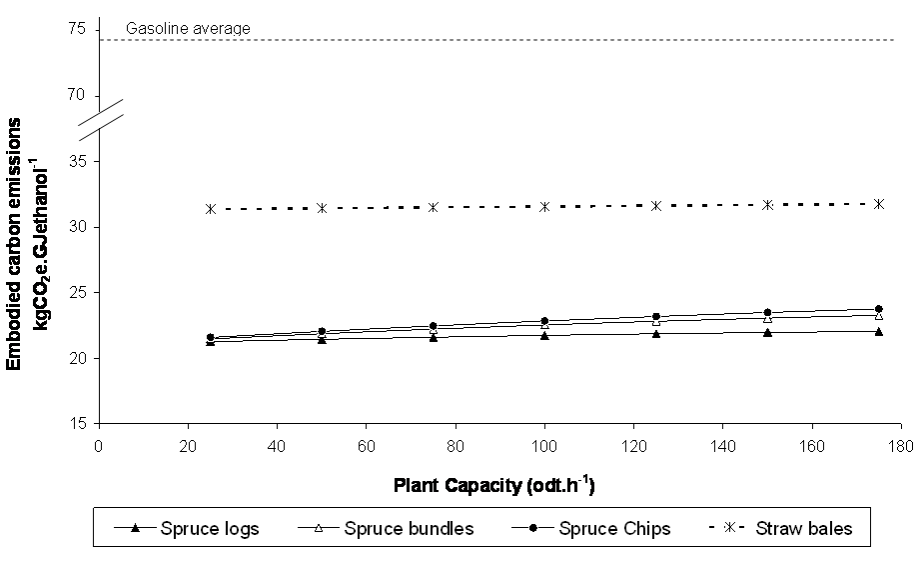
**The variation in supply chain greenhouse gas emissions owing to increased transport distances due to increasing plant capacity**. The example shown is for the enzymatic hydrolysis process, all fossil carbon emissions are allocated to ethanol and solid fuel in proportion to their energy content.

A large plant requires feedstocks to be gathered from a larger area, thereby entailing greater transport emissions. To show the effect of increasing plant capacity on GHG emissions, base-case supply chains were modified to include the logistics model described above. Results for the enzymatic hydrolysis conversion process are shown in Figure 7. It can be seen that the difference between emissions for a small (10 odt.h^-1^) plant and a large (150 odt.h^-1^) plant is only around 2 to 10%. So, while there is a trade-off between minimising transport emissions, obtaining economies of scale, and possibly improvements in process integration at the plant level, the GHG consequences of this trade-off are minimal. Although not shown, similar relationships can be demonstrated for the other chains.

### Consequential impacts: indirect land-use change and fertiliser replacement

**Figure 8:**
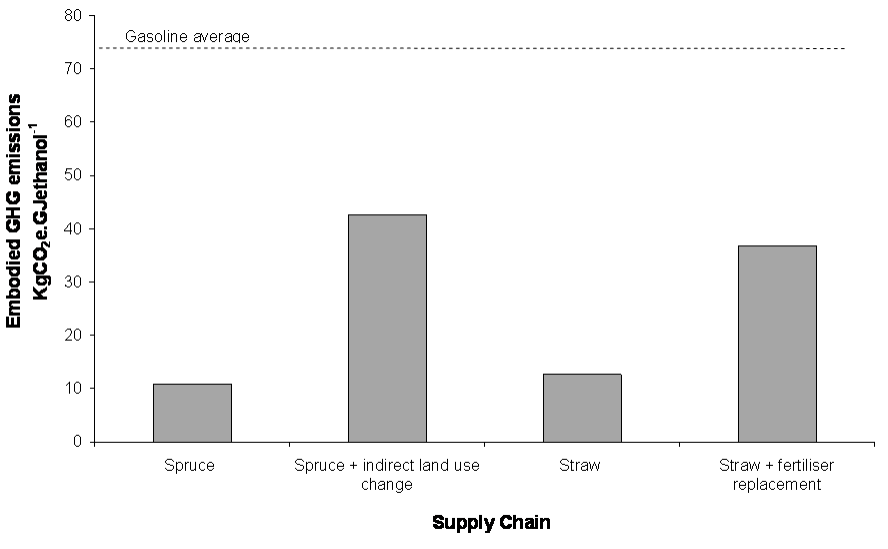
**Greenhouse gas emissions for supply chains including consequential impacts (indirect land use change and fertilizer replacement)**. The example shown assumes an enzymatic hydrolysis process, EU 27 electricity, and that emissions are allocated to ethanol and surplus solid fuel in proportion to their energy content.

If the consequential impacts due to land-use change and fertiliser replacement are included, supply chain emissions could increase dramatically. This is illustrated in Figure 8 for the softwood and straw chains (again, assuming that emissions are allocated to ethanol and surplus solid fuel). In this instance, for softwood, including indirect land-use change effects reduces the GHG saving from 70% to 27%, and for straw, introducing fertiliser replacement reduces the GHG saving from 56% to 24%. If co-products are not taken into account, or an allocation methodology that presents the process from a less advantageous perspective is used, the detrimental effect of including these impacts appears even greater.

The ILUC estimate for wood included here is somewhat crude and may be overly pessimistic; nevertheless, it is clear that indirect land-use change could have an important impact if demand for lignocellulosic feedstocks were to indirectly increase competition for land. In the case of increasing the utilisation of waste products such as straw, the inclusion of fertiliser replacement has a similar effect. In this case, however, it might be argued that a portion of the consequential impacts should be attributed to the primary product, the grain. Ultimately this is a subjective judgement.

### Sensitivity analysis

The relationships shown above clearly illustrate that emission estimates are influenced by a great number of assumptions. Testing the stability of the model output against variation in these assumptions is important to identify which have the greatest influence. In this analysis, the testing has been broken down into two steps.

In the first step, an elasticity analysis was conducted on each of the GHG emission factor parameters feeding into the model. The elasticity of a result with respect to an input parameter is defined as the ratio of the percentage change of the result to the percentage change in the parameter. A small change, much less than 1, denotes an inelastic parameter, that is, one that is forgiving of small uncertainties, whereas an elasticity close to 1 shows that the parameter has a greater influence on the model result and indicates that a more accurate input is required.

In the second step, the parameters identified as important were varied over a range of values and the change in results recorded. The outcome is presented graphically in the form of a spider diagram showing the change in the result as a function of the percentage change in each parameter.

The elasticity of the kgCO_2_e.GJ^-1 ^metric was calculated with respect to all emission parameters and for all the supply chains. For clarity, only parameters with elasticity greater than 0.01 are shown; these are listed in Table 7[39,40].

**Table 7:** Emission factor parameters included in the sensitivity analysis.

**Parameter**	**Unit**	**Base-case value.unit^-1^**	**% Variation relative to base-case**		**Remark**
				
			**Min**	**Max**	
Softwood production transport and size reduction		46.0	0.5	1.9	Range reflects the difference between high and low estimates for softwood emissions, excluding land-use change
	kgCO_2_e.odt^-1^					
Straw production and transport		2.6	0.5	2	This range is illustrative only and reflects variation around the lower estimate for straw

Enzyme production	kgCO_2_e.kg protein^-1^	9.55	0.1	1.4	Range reflects the carbon intensity of enzyme manufacture using Swedish or UK electricity relative to EU 27

Process electricity purchased	kgCO_2_e.MWh^-1^	340.8	0.1	1.4	Range reflects the carbon intensity of Swedish and UK electricity relative to EU 27

H_2_SO_4_^a^		0.5	0.5	2	A range of -50% to +100% was considered sufficient
					to cover uncertainties in production emissions
NaOH^b^		0.58			
					
SO_2_^a^	kgCO_2_		0.641			
	e.kg^-1^				
(NH4)2PO4^c^		1.67			
					
MgSO_4_^.^7 H_2_O^d^		0.56			

^a^Boustead database [38]. ^b^DEAM database [39]. ^c^Estimated from mass proportion of NH_3 _with H_3_PO_4 _(reported in DEAM database). ^d^Reported in GREET 1.8, Ag_Inputs!AA43:AA52 – sum of production and process emissions, assumed to be the same as CaCO_3_.

**Figure 9:**
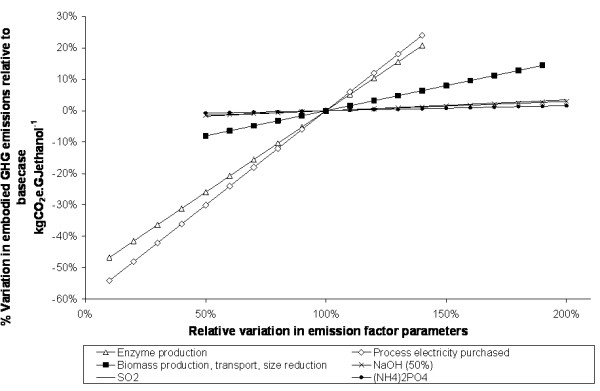
**The sensitivity of supply chain greenhouse gas performance to variations in emission factor assumptions – softwood enzymatic process**. The example shown assumes EU27 electricity, and that emissions are allocated to ethanol and solid fuel in proportion to their energy content.

**Figure 10:**
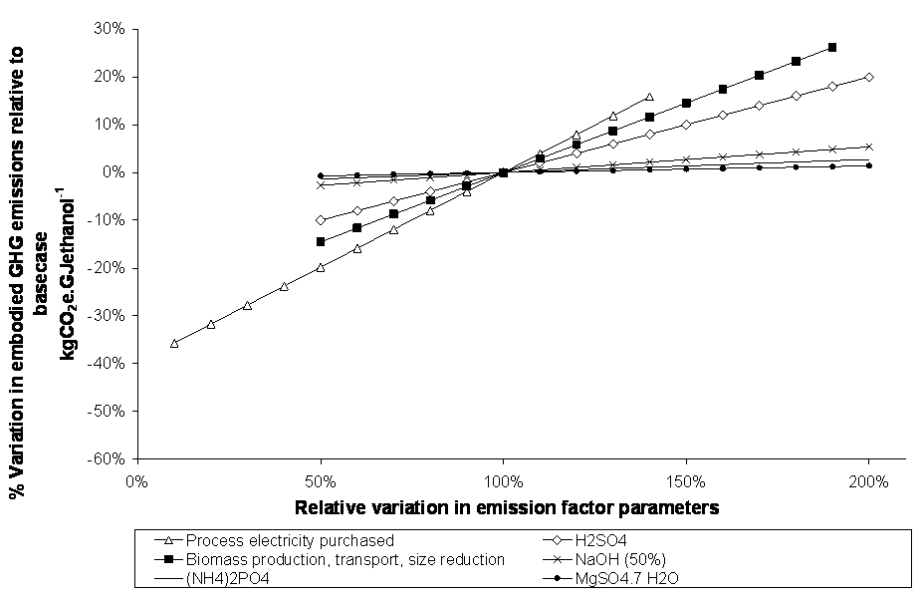
**The sensitivity of supply chain greenhouse gas performance to variations in emission factor assumptions – softwood dilute acid process**. The example shown assumes EU27 electricity, and that emissions are allocated to ethanol and solid fuel in proportion to their energy content.

The results of the sensitivity analysis are similar for softwood and straw chains. Illustrative spider diagrams showing the results for the softwood enzymatic and dilute acid processes are shown in Figures 9 and 10. For the examples shown, all parameters are positively correlated. The parameters with the greatest influence are the emissions factors for electricity, enzyme manufacture, biomass production, and, in the case of the dilute acid process, sulphuric acid. Other chemical inputs have little impact.

## Conclusion

The model described here has been used to characterise and compare simplified LE supply chains applicable to Europe. Using this model it has been possible to identify which factors are most important in determining GHG emission savings and to compare a range of supply chain configurations. The impact of selecting alternative allocation methodologies on the interpretation of the results was also considered.

Quantifying GHG emissions from the production of cellulosic ethanol faces similar challenges to quantifying the emissions from conventional biofuels. There is limited empirical data, significant methodological variation, and results cannot easily be divorced from subjective interpretation. Nevertheless, although the precise level of emissions may be uncertain, the LE supply chains considered here all lead to GHG savings relative to gasoline irrespective of the allocation method applied. There is, however, one important caveat to this generalisation: the essentially unknown importance of consequential impacts, and, in particular, indirect land-use change. If LE production uses feedstocks that lead to indirect land-use change, the GHG performance of LE supply chains may be greatly reduced.

If the commercial deployment of LE technology is modest and does not exceed the carrying capacity of existing managed forestry, then indirect land-use change could reasonably be ignored. The problem for policy makers is that replacing a significant proportion of European transport fuel requires anything but a modest response. Indirect land-use change is therefore likely to remain firmly on the political and scientific agenda. Further analysis is needed, and it is important that this analysis is sensitive to the multi-scale nature of bio-energy systems and the anticipated level of deployment.

The debate about how impacts should be allocated to co-products is also unlikely to diminish. The UK Renewable Transport Fuel Obligation – arguably the most advanced framework for assessing the GHG benefit of biofuels – indicates that substitution is the preferred approach, but permits other approaches be considered where substitution is not practical. This flexibility has considerable advantages: for an individual plant the substitution methodology probably provides the best reflection of the situation on the ground, but for the purpose of policy formation, where it is necessary to forecast and generalise the environmental impact of multiple plants in multiple locations, allocating emissions on the basis of energy content is simpler and will yield a more consistent result.

Setting the issues of consequential impacts and allocation to one side, the most important factors affecting supply chain emissions are the use of electricity and the GHG emissions embodied in biomass production. For any operational assessment of GHG impacts that may be required as part of a reporting scheme, it will therefore be important for fuel producers to know the provenance of their feedstocks.

The enzymatic process entails greater GHG emissions than the dilute acid process owing to the large quantity of electricity consumed during enzyme manufacture, even though the dilute acid process has a somewhat lower ethanol yield. Co-locating ethanol production, electricity generation and enzyme production in a single facility may improve performance, particularly if this allows the number of energy intensive steps in enzyme production to be reduced, or if other process synergies are available. Determining the extent of these synergies requires a more detailed process comparison than the one presented here. What is clear, however, is that without these synergies, integrating enzyme production on site is simply equivalent to selecting a system expansion methodology to allocate environmental impacts in preference to one that apportions impacts to products and co-products.

While process integration provides scope to decrease electricity consumption and advances in microbiology have the potential to increase enzyme yield and efficacy, the emissions embodied in biomass production are potentially more difficult to reduce: improvements will ultimately depend on yield improvements and efficiency gains. Transport emissions, although often discussed in the literature, appear to be relatively unimportant.

If biofuels policy in the EU remains contingent on favourable environmental performance then the multi-scale nature of bioenergy supply chains presents a genuine challenge. LE holds promise for emission reductions, but maximising GHG savings will not only require efficient supply chain design but also a better understanding of the spatial and temporal factors which affect overall performance.

## List of abbreviations

C: capacity; DA: dilute acid conversion process; DAp: dilute acid conversion process incorporating pentose fermentation; EH: enzymatic conversion process; EHp: enzymatic conversion process incorporating pentose fermentation; FPU: filter paper unit; GHG: greenhouse gas; ILUC: indirect land-use-change; LCA: life cycle assessment; LE: lignocellulosic ethanol; NILE: New Improvements in Lignocellulosic Ethanol (An EU Framework Programme 7 project); NREL: National Renewable Energy Laboratory; odt: oven dry tonnes; SRC: short rotation coppice.

## Competing interests

The authors declare that they have no competing interests.

## Authors' contributions

RS designed and executed the study including model development, results analysis and drafting of the manuscript. AB conceived the study, and both AB and NS participated in the study design. All authors read, commented upon and approved the final manuscript.
